# Supplementary material for: Disordered breathing in a Pitt-Hopkins syndrome model involves Phox2b-expressing parafacial neurons and aberrant Nav1.8 expression
Source: Nat Commun. 2021 Oct 13;12:5962. doi: 10.1038/s41467-021-26263-2 (PMC8514575; doi:10.1038/s41467-021-26263-2)
Supplement: Supplementary file 1 — Supplementary Information [file 41467_2021_26263_MOESM1_ESM.pdf]

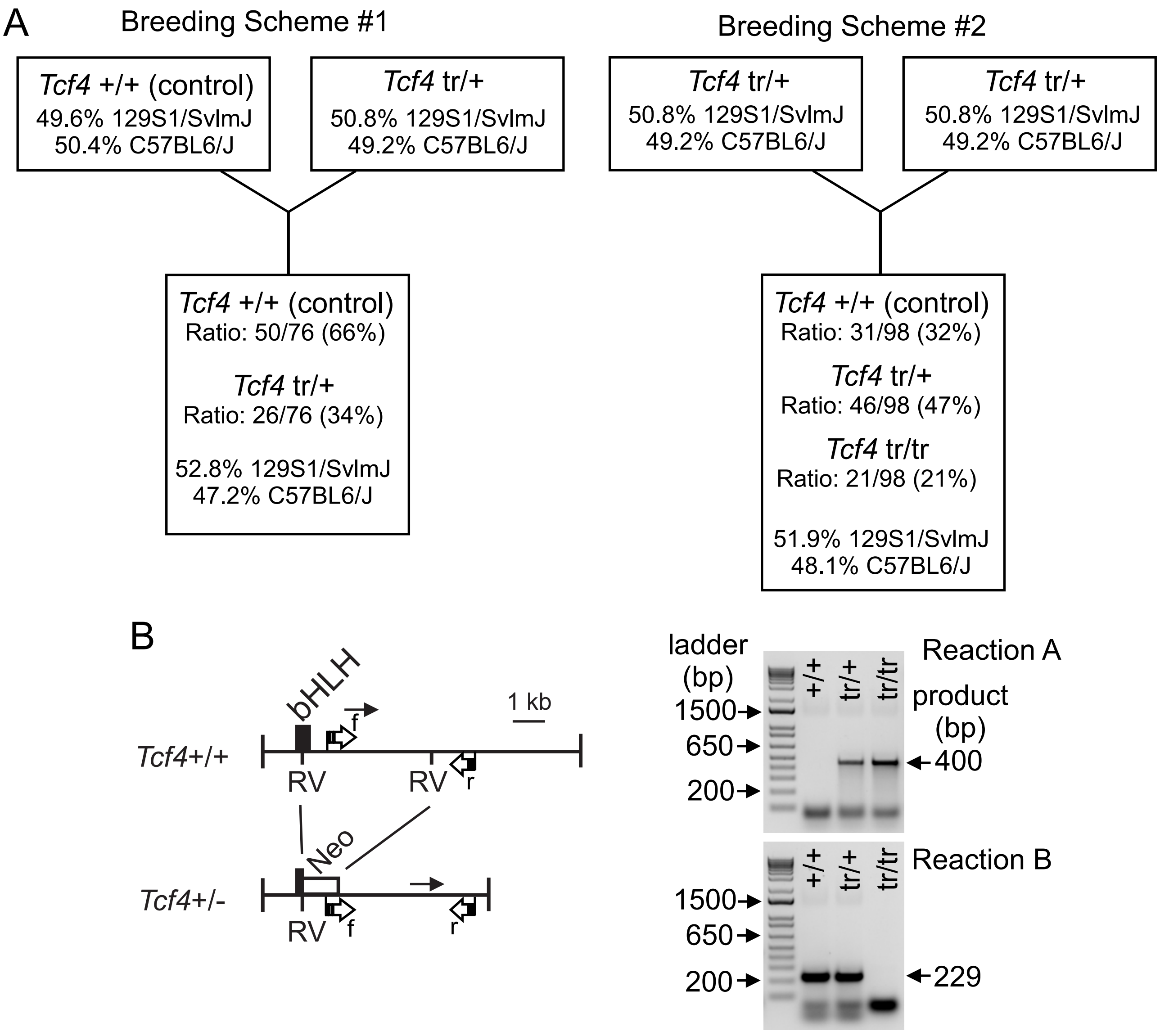

Supplementary Figure 1

Supplementary Figure 1: Breeding scheme and *Tcf4* truncation targeting strategy A, *Tcf4*$^{tr/+}$ were bred with themselves (scheme 2) or *Tcf4*$^{+/+}$ (scheme 1) to generate *Tcf4*$^{+/+}$, *Tcf4*$^{tr/+}$ and *Tcf4*$^{tr/tr}$ animals all on a similar background of 50% 129S1/SvlmJ and 50% C57BL6/J. The proportion of each background strain was determined by Genome scan analysis (JAX). **B** left, *Tcf4* targeting strategy redrawn from Zhuang et al[1] and published here with permission from the journal. Note that a large portion of the basic helix–loop–helix (BHLH) domain of *Tcf4* was replaced with a neomycin phosphotransferase (Neo) gene. Arrows designate gene orientation and location of forward (f) and reverse (r) primers. Right, agarose gels show detectable levels of wild type (expected size of 229 bp) and truncated (expected size of 400 bp) transcript for each genotype. Animals were genotyped once per reaction.

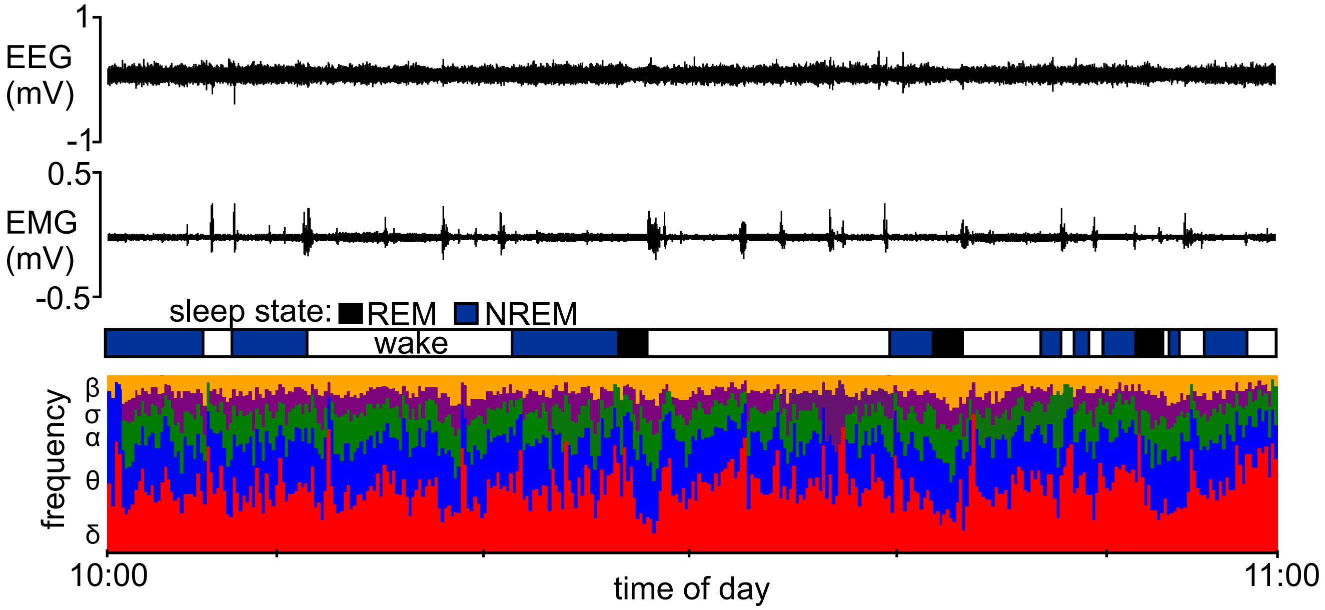

Supplementary Figure 2

Supplementary Figure 2: *Tcf4*$^{tr/+}$ mice do not show overt seizures or a seizure-like brain activity Traces of electrocorticogram (ECoG) activity, electromyography (EMG) activity and corresponding spectrogram (bottom) show that *Tcf4*$^{tr/+}$ mice (n=6 mice) do not exhibit characteristic seizure-like ECoG activity (high amplitude poly-spike activity) during 60-minute recordings under room air conditions. The ECoG and EMG signals were also used to identify awake, non-REM (NREM) and paradoxical/REM sleep periods; non-REM was identified by an increase in delta power and a lack of muscle activity and REM sleep was identified by a decrease in delta power and concurrent increase in beta/theta power but with minimal EMG activity. Frequency ranges of the EcoG signal are defined as follows: delta, δ (0.5-4 Hz, red), theta, θ (4-8 Hz, blue), alpha, α (8-12 Hz, green), σ, sigma (12-16 Hz, purple), beta, β (16-24 Hz, yellow). Note that *Tcf4*$^{tr/+}$ mice also did not show obvious seizure like behavior at any point during these experiments.

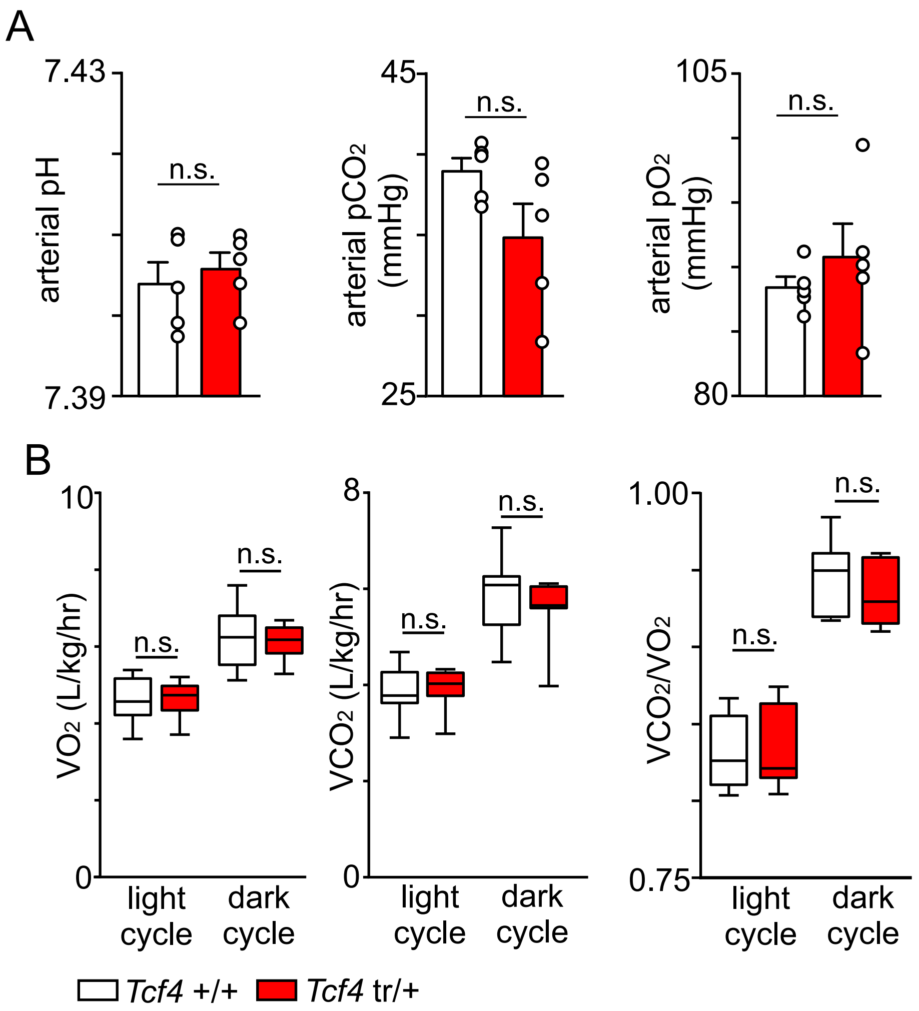

Supplementary Figure 3

Supplementary Figure 3: Metabolic and blood gas parameters are similar in control and *Tcf4*$^{tr/+}$ mice A, Under room air conditions, *Tcf4*$^{+/+}$ and *Tcf4*$^{tr/+}$ mice (N = 5 animals/genotype) show similar arteriole pH (T_8=0.5518, p>0.05, data are presented as mean values ± SEM), pCO_2 (T_8=1.835, p>0.05, data are presented as mean values ± SEM), and pO_2 (T_8=1.263, p>0.05, data are presented as mean values ± SEM). **B**, Oxygen consumption (VO_2) (light, T_{12}=0.1356, p>0.05; control- minima: 3544, 25% CI: 4145, median: 4567, 75% CI: 5243, maximum: 5448, *Tcf4*$^{tr/+}$- minima: 3655, 25% CI: 4271, median: 4739, 75% CI: 5054, maximum: 5255; dark, T_{12}=0.3452, p>0.05, control- minima: 5062, 25% CI: 5454, median: 6242, 75% CI: 6881, maximum: 7659, *Tcf4*$^{tr/+}$- minima: 5226, 25% CI: 5751, median: 6183, 75% CI: 6572, maximum: 6752), CO_2 production (VCO_2) (light, T_{12}=0.9184, p>0.05, control- minima: 2855, 25% CI: 3572, median: 3784, 75% CI: 4329, maximum: 4745, *Tcf4*$^{tr/+}$- minima: 2939, 25% CI: 3714, median: 4028, 75% CI: 4319, maximum: 4330 ; dark, T_{12}=0.6248, p>0.05, control- minima: 4428, 25% CI: 5197, median: 6080, 75% CI: 6320, maximum: 7320, *Tcf4*$^{tr/+}$- minima: 3928, 25% CI: 5545, median: 5657, 75% CI: 6108, maximum: 6133), and the respiratory exchange ratio (light, T_{12}=0.09722, p>0.05, control- minima: 0.8022, 25% CI: 0.8086, median: 0.8262, 75% CI: 0.8572, maximum: 0.8681, *Tcf4*$^{tr/+}$- minima: 0.8031, 25% CI: 0.8133, median: 0.8211, 75% CI: 0.8652, maximum: 0.8754; dark, T_{12}=0.1297, p>0.05, control- minima: 0.8720, 25% CI: 0.9172, median: 0.9478, 75% CI: 0.9549, maximum: 0.9864, *Tcf4*$^{tr/+}$- minima: 0.9087, 25% CI: 0.9134, median: 0.9294, 75% CI: 0.9602, maximum: 0.9622) were similar between control (N = 7 animals) and *Tcf4*$^{tr/+}$ (N = 7 animals) mice during the dark/active and light/inactive states. Baseline metabolic activity and blood gas values were compared using an unpaired t-test.

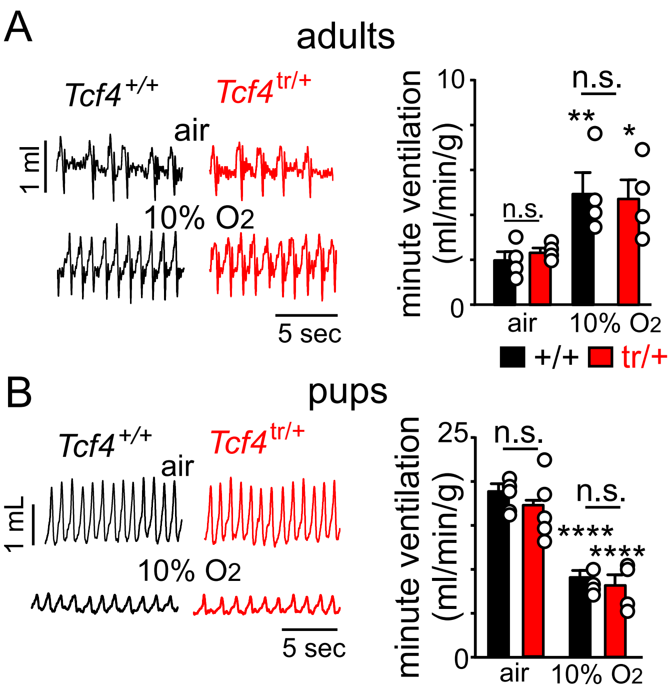

Supplementary Figure 4

Supplementary Figure 4: Neonatal and adult *Tcf4*$^{tr/+}$ mice show a normal ventilatory response to acute hypoxia A, Traces of respiratory activity and summary data (N=4 mice/genotype) from adult (47-48 days of age) control and *Tcf4*$^{tr/+}$ mice show that both genotypes respond to hypoxia (10% O_2, balance N_2) with similar increases in respiratory output ($F_{1,3}$=0.03643, p>0.05, data are presented as mean values ± SEM). **B**, Traces of respiratory activity and summary data (N=5 mice/genotype) from (11 days of age) control and *Tcf4*$^{tr/+}$ mouse pups show that hypoxia (10% O_2, balance N_2) suppresses respiratory output by proportional amounts in both genotypes ($F_{1,4}$=0.6860, p>0.05, data are presented as mean values ± SEM). These results suggest that peripheral chemoreception is normal at two developmental time points. One symbol= p <0.05, two symbols = p <0.01, three symbols = p <0.001, four symbols = p <0.0001 (two-way RM-ANOVA with Tukey's multiple comparison test).

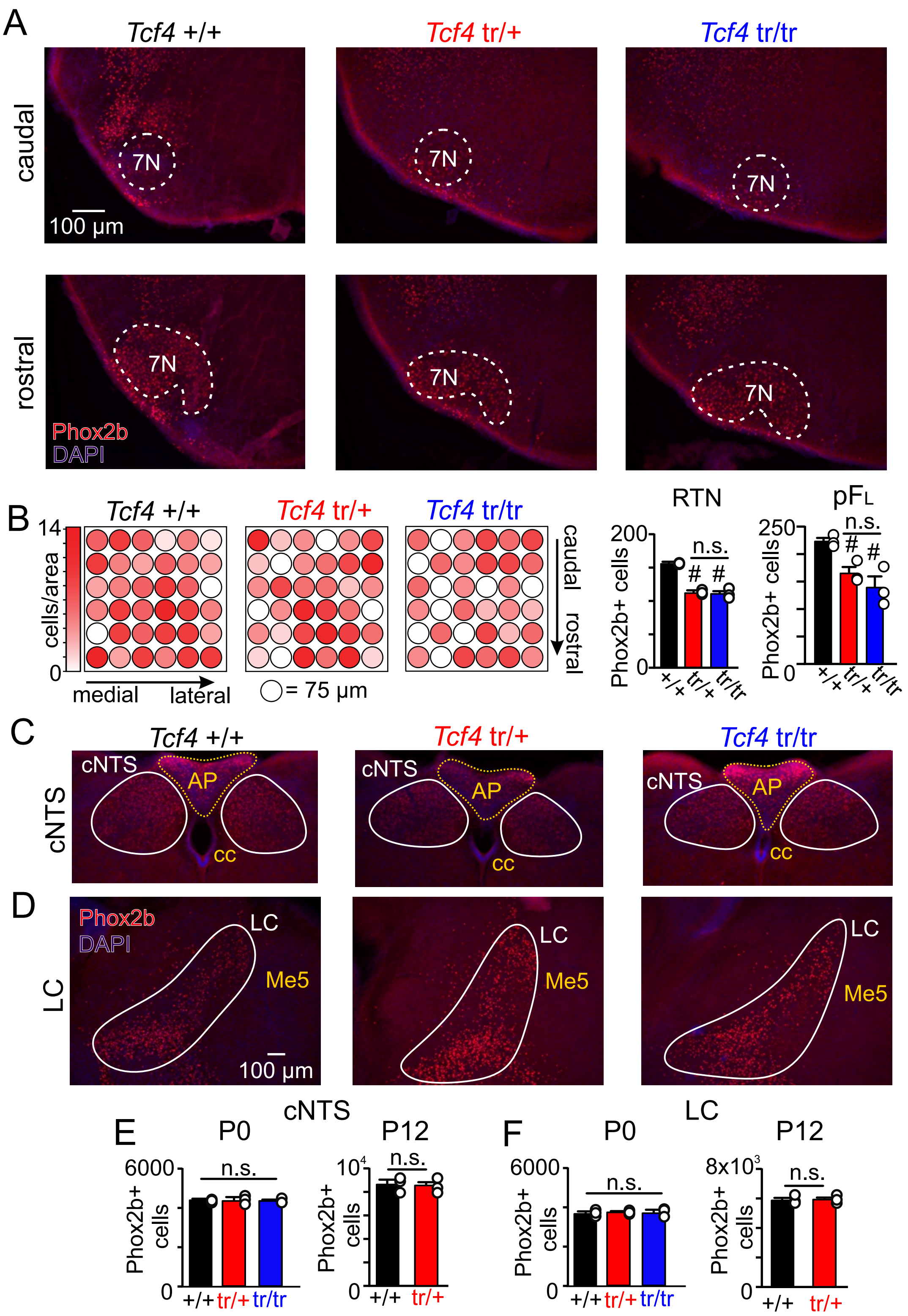

Supplementary Figure 5

Supplementary Figure 5. Phox2b+ parafacial neurons are selectively disrupted early in development in *Tcf4*$^{\mathbf{tr/+}}$ mice A, Photomicrographs of coronal sections from newborn *Tcf4*$^{+/+}$, *Tcf4*$^{tr/+}$, and *Tcf4*$^{tr/tr}$ mouse pups show Phox2b-immunoreactivity (Phox2b-IR, red) in the caudal (top) and rostral (bottom) parafacial region. The number of Phox2b+-labeled cells within ~75 µm of the ventral surface was tabulated for each genotype for 450 µm along the ventral surface starting just medial to the trigeminal nucleus, the lateral most 75 µm was considered the pF_L. **B,** Summary data (n=3 mice/genotype) show the distribution of Phox2b-IR soma across the caudal to rostral (y axis; 6 slices total per animal) and medial to lateral (x axis) extent of the parafacial region. Right, bar graphs show that Phox2b labeling is significantly diminished in RTN and pF_L parafacial regions ($F_{2,6}$=10.62, p=0.0107, data are presented as mean values ± SEM). **C-D,** Photomicrographs of coronal sections from newborn *Tcf4*$^{+/+}$, *Tcf4*$^{tr/+}$, and *Tcf4*$^{tr/tr}$ pups show similar levels of Phox2b-IR (Phox2b-IR, red) in the cNTS (**C**) and LC (**D**). **E-F,** summary data show that Phox2b labeling in the cNTS (**E**; P12: 6 slices per animal/genotype, T_4=0.3499, p>0.05; P0: 4 slices per animal/genotype, $F_{2,6}$=0.2422, p>0.05) and LC (**F**; P12: 4 slices per animal/genotype, T_4=0.2427, p>0.05; P0: 2 slices per animal/genotype, $F_{2,6}$=0.0840, p>0.05) is similar between genotypes at two developmental time points (all data are presented as mean values ± SEM). #, different between genotypes (two way ANOVA with Tukey's post-hoc multiple comparison test). 7N= facial nucleus, cNTS= caudal nucleus tractus solitarius, AP= area postrema, cc=central canal, LC= locus coeruleus, Me5= mesencephalic trigeminal nucleus

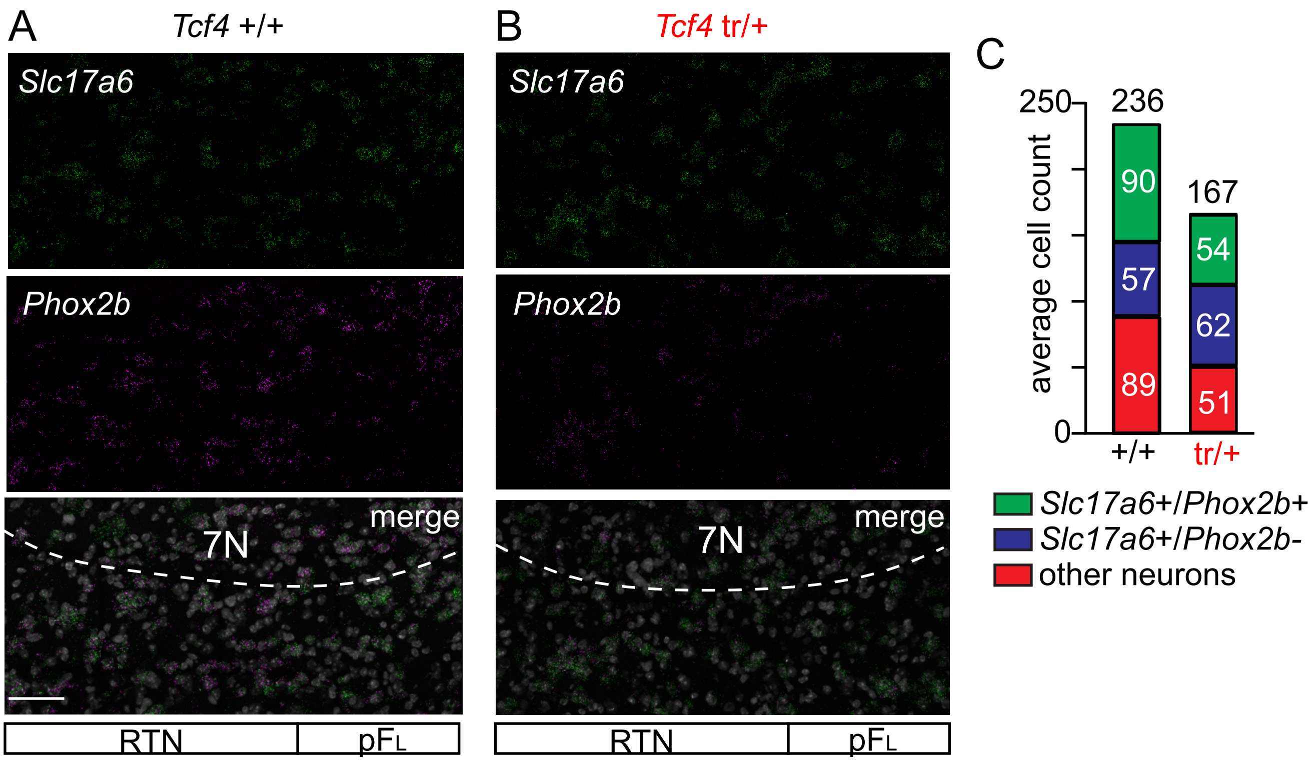

Supplementary Figure 6

Supplementary Figure 6. Total numbers of glutamatergic parafacial neurons is maintained in *Tcf4*$^{tr/+}$ mice Photomicrographs of coronal sections (n=3 animals, age matched and mixed sex) containing the caudal parafacial region from 12 day old control (**A**) and *Tcf4*$^{tr/+}$ (**B**) mice show *Slc17a6* (gene encoded vesicular glutamate transporter 2; Vglut2) transcript in green (top), *Phox2b* transcripts in magenta (middle), and merged (bottom). **C**, summary data (n=3, age matched and mixed sex) shows that of the averaged 147 *Slc17a6*+ neurons identified in the parafacial region from control mice, 90 cells also showed Phox2b labeling (61%, green). However, of the averaged 116 *Slc17a6*+ neurons identified in the parafacial region from *Tcf4*$^{tr/+}$ mice, 54 cells showed Phox2b co-labeling (46%, green). Average cell counts in both genotypes indicate a 21% loss of *Slc17a6*+ cells in *Tcf4*$^{tr/+}$ mice (14% loss of cells expressing both *Slc17a6*+ and *Phox2b*+, indicated in green, and 7% loss of *Slc17a6*+ cells only, indicated in blue).

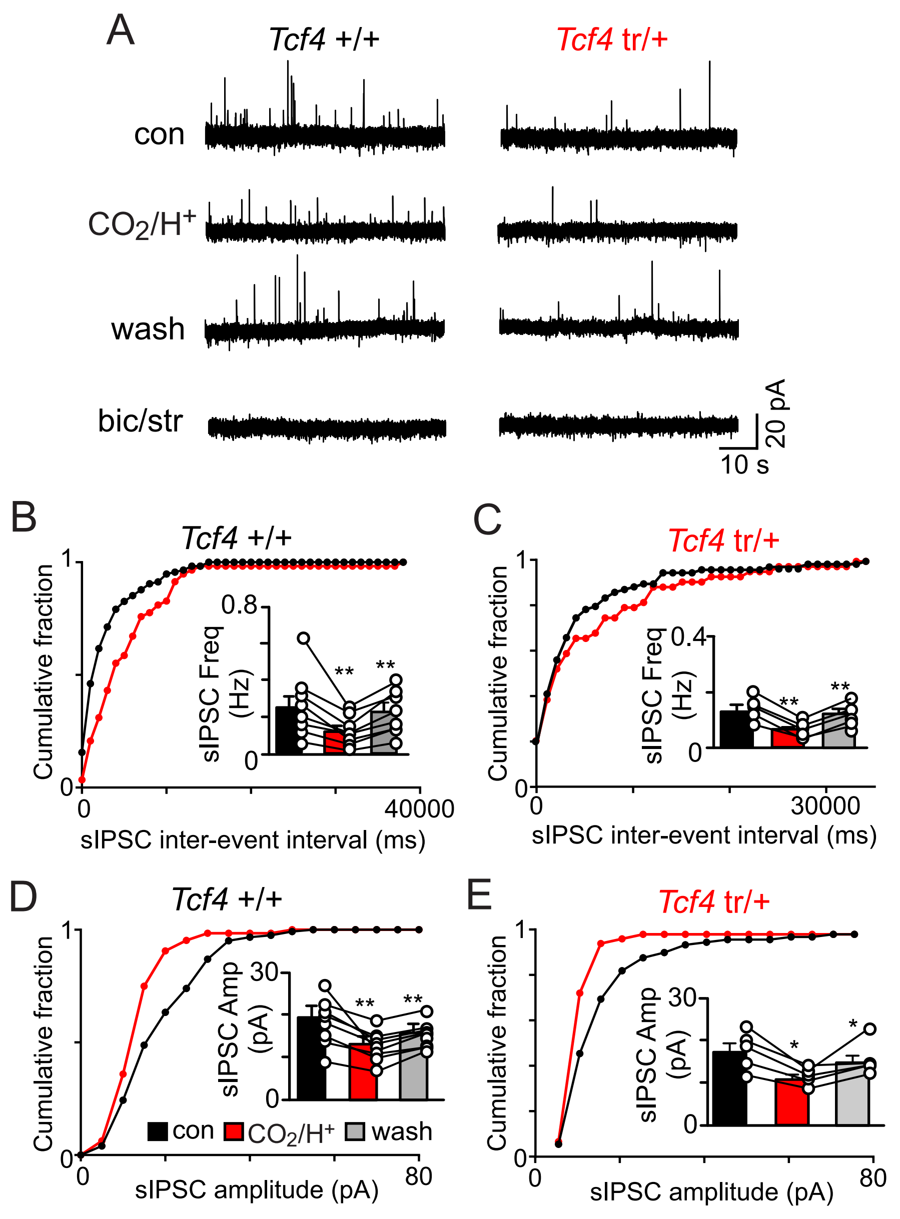

Supplementary Figure 7

Supplementary Figure 7. CO_2/H^+-dependent inhibitory synaptic modulation of chemosensitive RTN neurons is similar between genotypes A, traces of holding current (Ihold = 0 mV) from an RTN chemoreceptor in slices from *Tcf4*$^{+/+}$ and *Tcf4*$^{tr/+}$ mice shows spontaneous inhibitory postsynaptic currents (sIPSC) under control conditions and during exposure to 10% CO_2 or bicuculline (10 µM) and strychnine (2 µM). **B-C**, summary (N=8 cells control; N=5 cells *Tcf4*$^{tr/+}$) cumulative distribution plots of sIPSC inter-event interval (bin size: 250 ms) and bar graphs of mean sIPSC frequency under each experimental condition shows that RTN neurons from both genotypes have similar sIPSC frequency under control conditions (T_{12}=1.407, p>0.05, data are presented as mean values ± SEM) and during high CO_2 (T_{12}=1.576, p>0.05, data are presented as mean values ± SEM). **D-E**, cumulative distribution plots of sIPSC amplitude (bin size: 5 pA) and bar graphs of mean sIPSC amplitude under each condition show that amplitude of sIPSC was similar between genotypes under control conditions (T_{12}=0.5989, p>0.05, data are presented as mean values ± SEM) and during exposure to high CO2 (T_{12}=1.028, p>0.05, data are presented as mean values ± SEM). Statistics indicated in figure was analyzed by one-way RM ANOVA followed by Tukey multiple comparison test. In text statistics comparing control and *Tcf4*$^{tr/+}$ mice use unpaired t-tests. *, difference from control at p <0.05 (one symbol) or p <0.01 (two symbols).

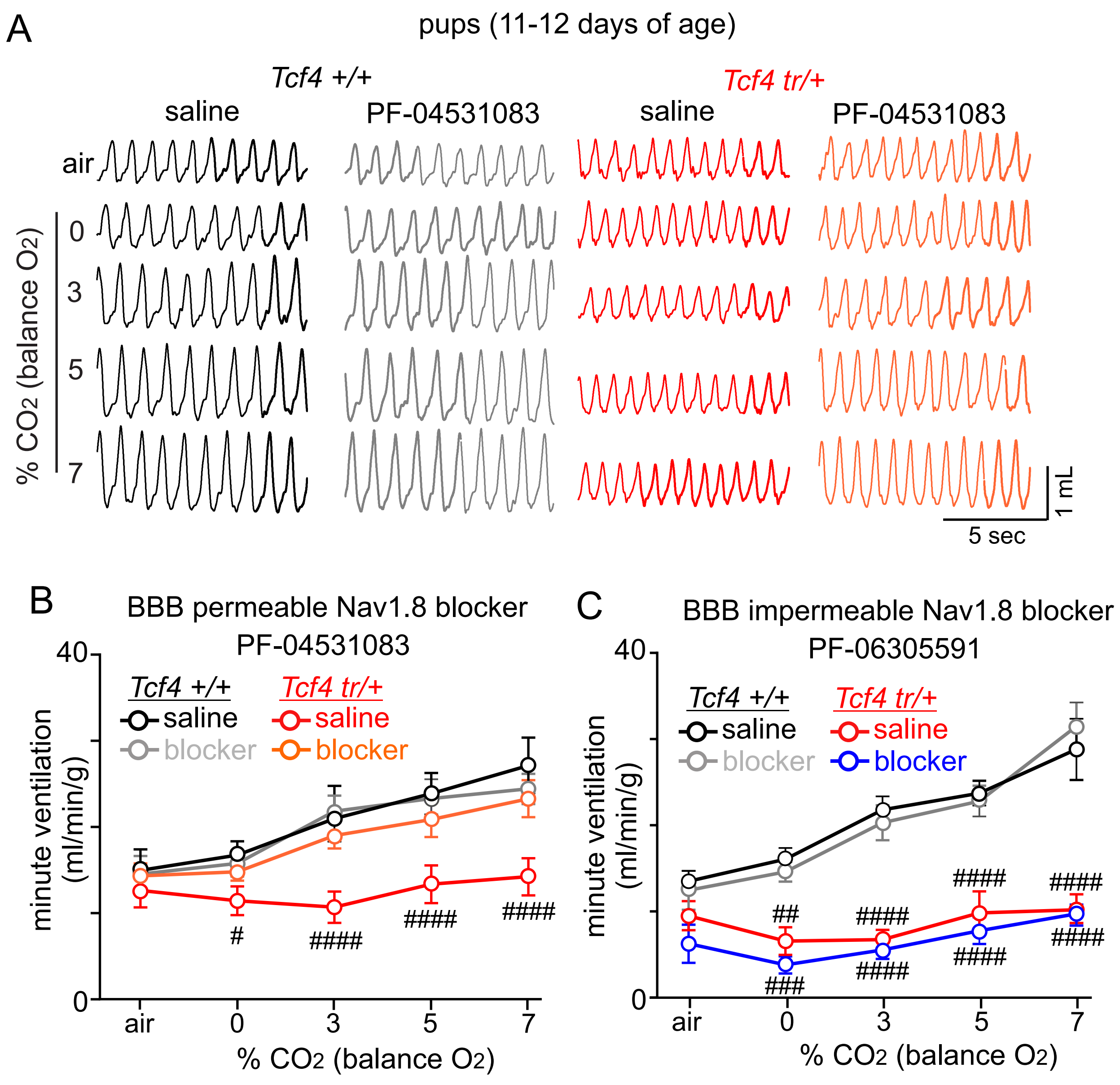

Supplementary Figure 8

Supplementary Figure 8. Blockade of central Nav1.8 channels fully rescued chemoreception deficits in *Tcf4*$^{tr/+}$ pups A, Respiratory activity was characterized in *Tcf4*$^{+/+}$ pups (11-12 days old) ~1.5 hours after systemic (I.P) administration of saline (30 µL, black) or PF-04531083 (40 mg/kg, grey) and in *Tcf4*$^{tr/+}$ mouse pups after systemic (I.P.) administration of saline (red) or PF-04531083 (orange). **A**, traces of respiratory activity from saline or PF-04531083 treated *Tcf4*$^{+/+}$ and *Tcf4*$^{tr/+}$ mice during exposure to room air, 100% O_2 and 3-7% CO_2 (balance O_2). **B,** summary plots of minute ventilation show that saline treated *Tcf4*$^{tr/+}$ pups lack the ability to increase respiratory output during high CO_2 ($F_{1,4}$=43.19, p=0.0028, data are presented as mean values ± SEM). However, this chemoreceptor deficit was fully rescued by systemic administration of PF-04531083 ((0-7% CO_2 slope: 0.59 ± 0.08 saline vs. 0.79 ± 0.10 PF-04531083; p=0.0314, data are presented as mean values ± SEM) to a level not different from *Tcf4*$^{+/+}$ mice (0-7% CO_2 slope: 1.11 ± 0.1; p>0.05, data are presented as mean values ± SEM). **C**, summary plots of minute ventilation show that systemic application of PF-06305591 (2 mg/kg) minimally effected respiratory activity *Tcf4*$^{+/+}$ (grey) and *Tcf4*$^{tr/+}$ pups (blue) (0-7% CO_2 slope: 0.56 ± 0.06 saline vs. 0.50 ± 0.09 PF-06305591; p>0.05, data are presented as mean values ± SEM). *Tcf4*$^{+/+}$ pups showed similar levels of respiratory activity following administration of saline and PF-04531083 ($F_{1,4}$=2.088, p>0.05, data are presented as mean values ± SEM). #, different between genotypes (two -way RM-ANOVA with Tukey's multiple comparison test). One symbol= p <0.05, two symbols = p <0.01, three symbols = p <0.001, four symbols = p <0.0001

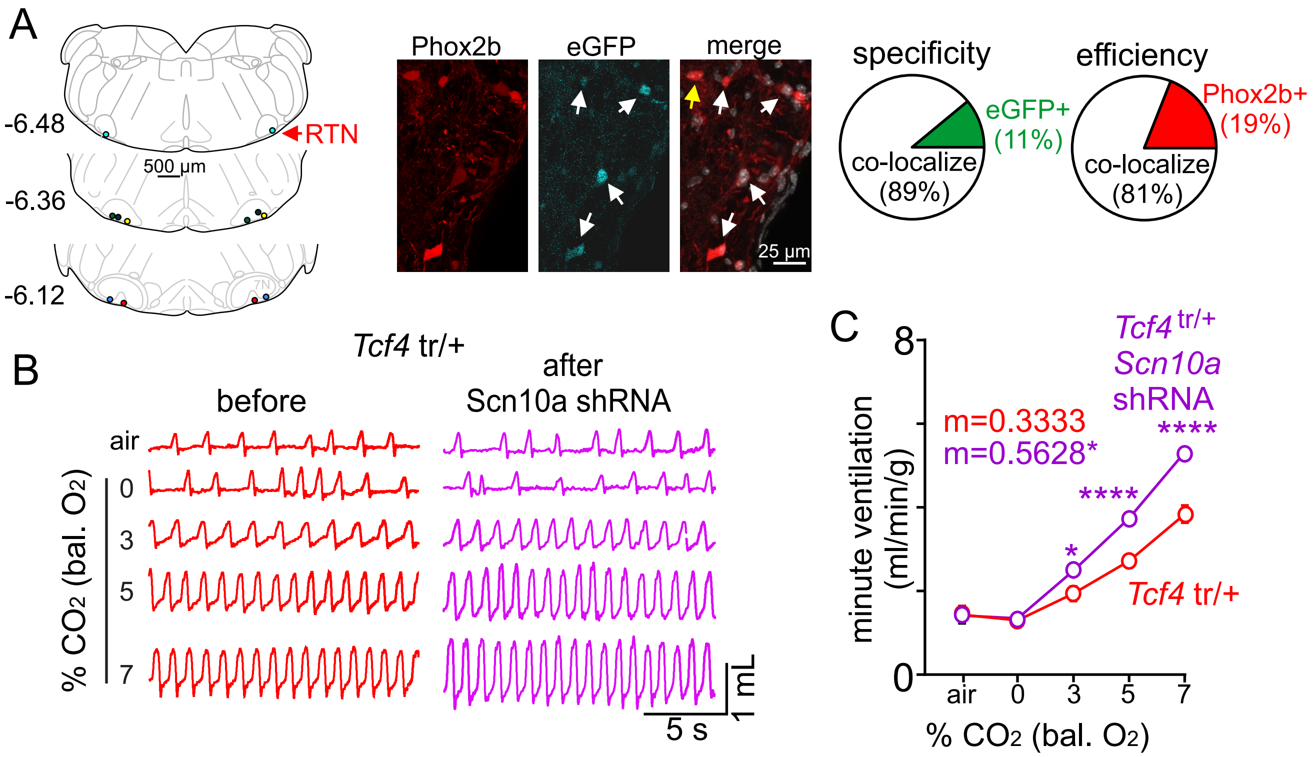

Supplementary Figure 9

Supplementary Figure 9. RTN chemoreceptor specific shRNA knock down of *Scn10a* improved the CO_2 ventilatory response in *Tcf4*$^{tr/+}$ mice **A**, computer-assisted plots show centers of bilateral AAV2-PRSx8-eGFP-mScn10a-shRNAmir injections in *Phox2b*^{Cre}::Ai14::*Tcf4*$^{tr/+}$ mice (n=6 mice, mixed sex). The numbers to the left of each section indicates the relative position of each slice behind bregma. Images and corresponding summary data show that two weeks after injection 89% of viral mediated GFP labeling (cyan) colocalized with Phox2b reporter labeling (red) and 81% of Phox2b expressing neurons in this region were transfected and express GFP (white area of pie charts indicates co-localization of GFP signal with TdTomato reporter; green area is GFP signal in the absence of TdTomato; red is TdTomato in the absence of GFP signal). **B**, traces of respiratory activity from *Phox2b*^{Cre}::Ai13::*Tcf4*$^{tr/+}$ mice before (red) and two weeks after (magenta) bilateral viral injections. **C**, summary plots of minute ventilation show that *Scn10a* shRNA improved CO2-dependent respiratory output ($F_{1,5}$=16.84, p=0.0093, data are presented as mean values ± SEM). Slopes between 0% and 7% CO_2 were compared by analysis of covariance (ANCOVA). *, different between genotypes (two-way RM-ANOVA with Tukey's multiple comparison test; one symbol p < 0.01, two symbols p<0.01; three symbols p<0.001, four symbols p<0.0001).

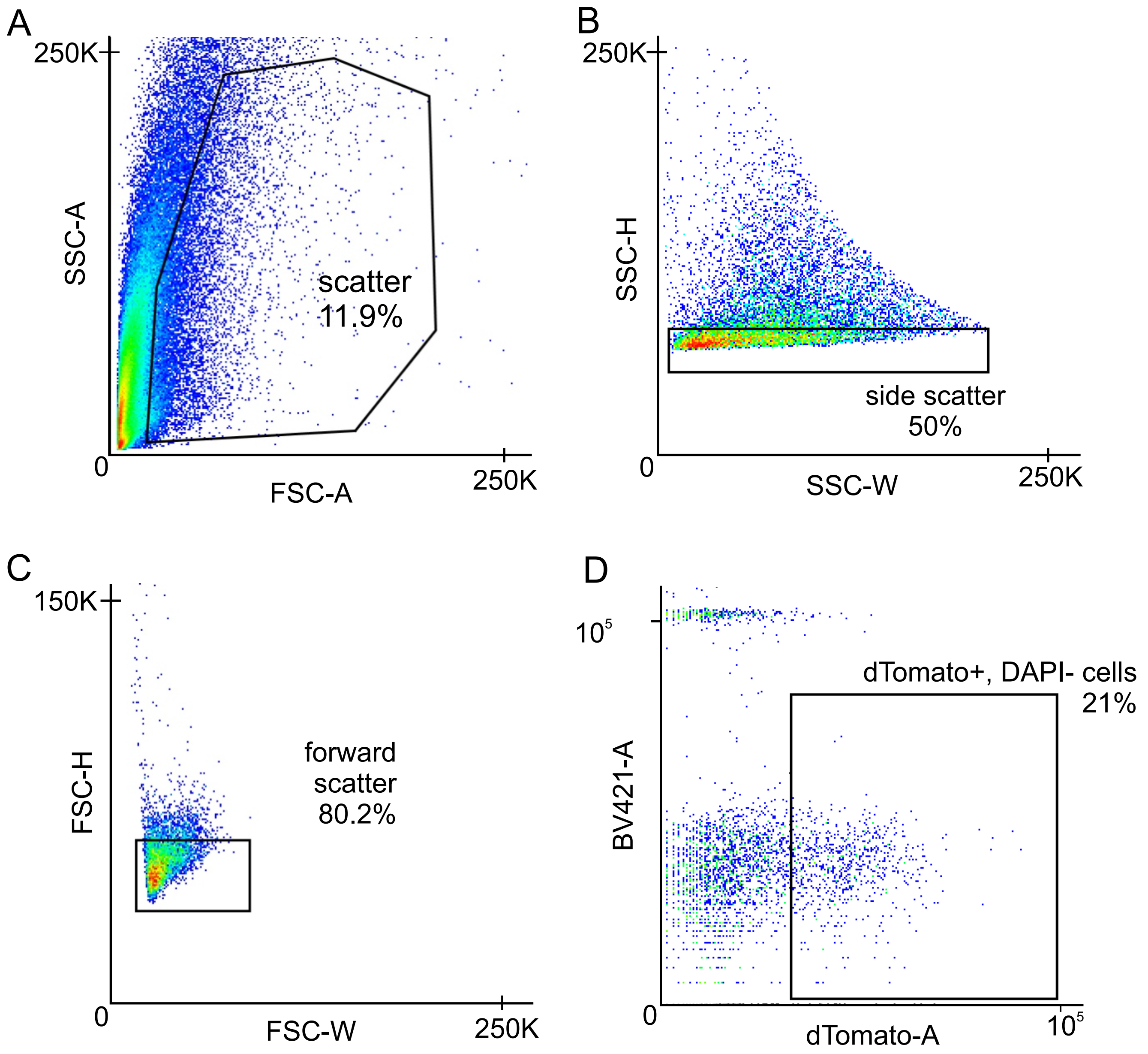

Supplementary Figure 10

Supplementary Figure 10: FACS gating strategy for sorting TdTomato fluorescent cells from the RTN A, Scatter graph gating out debris from the sample. **B**, Side scatter graph to gate for complexity/doublets. **C**, Forward scatter graph to gate for cell size. **D**, Scatter graph for DAPI and TdTomato. Cells were gated for positive TdTomato and low DAPI signal.

Supplementary Table 1: Primers used for *Tcf4* mouse line

	Reaction A (splice variant, 5' to 3')	Reaction B (control, 5' to 3')
**Forward**	AGCGCGAGAAAGGAACGGAGGA	
**Reverse**	CCAGAAAGCGAAGGAGCA	GGCAATTCTCGGGAGGGTGCTT

Supplementary References

1 Zhuang, Y., Cheng, P. & Weintraub, H. B-lymphocyte development is regulated by the combined dosage of three basic helix-loop-helix genes, E2A, E2-2, and HEB. *Mol Cell Biol* **16**, 2898-2905, doi:10.1128/mcb.16.6.2898 (1996).
