# Supplementary material for: Disordered breathing in a Pitt-Hopkins syndrome model involves Phox2b-expressing parafacial neurons and aberrant Nav1.8 expression
Source: Nat Commun. 2021 Oct 13;12:5962. doi: 10.1038/s41467-021-26263-2 (PMC8514575; doi:10.1038/s41467-021-26263-2)
Supplement: Supplementary file 3 — Reporting summary [file 41467_2021_26263_MOESM3_ESM.pdf]

nature portfolio

Corresponding author(s): Daniel K. Mulkey
Last updated by author(s): Sep 2, 2021

Reporting Summary

Nature Portfolio wishes to improve the reproducibility of the work that we publish. This form provides structure for consistency and transparency in reporting. For further information on Nature Portfolio policies, see our Editorial Policies and the Editorial Policy Checklist.

Statistics

For all statistical analyses, confirm that the following items are present in the figure legend, table legend, main text, or Methods section.

n/a	Confirmed	
☐	☒	The exact sample size (n) for each experimental group/condition, given as a discrete number and unit of measurement
☐	☒	A statement on whether measurements were taken from distinct samples or whether the same sample was measured repeatedly
☐	☒	The statistical test(s) used AND whether they are one- or two-sided<br>*Only common tests should be described solely by name; describe more complex techniques in the Methods section.*
☒	☐	A description of all covariates tested
☐	☒	A description of any assumptions or corrections, such as tests of normality and adjustment for multiple comparisons
☐	☒	A full description of the statistical parameters including central tendency (e.g. means) or other basic estimates (e.g. regression coefficient) AND variation (e.g. standard deviation) or associated estimates of uncertainty (e.g. confidence intervals)
☐	☒	For null hypothesis testing, the test statistic (e.g. F, t, r) with confidence intervals, effect sizes, degrees of freedom and P value noted<br>*Give P values as exact values whenever suitable.*
☒	☐	For Bayesian analysis, information on the choice of priors and Markov chain Monte Carlo settings
☒	☐	For hierarchical and complex designs, identification of the appropriate level for tests and full reporting of outcomes
☒	☐	Estimates of effect sizes (e.g. Cohen's d, Pearson's r), indicating how they were calculated

Our web collection on statistics for biologists contains articles on many of the points above.

Software and code

Policy information about availability of computer code

Data collection	All of the commericial software (EthoVision 13, Ponemah 5.32, ClampEx 11.0.3, LabChart 8, Oxymax v5.54, Keyence BZ-X700 Acquisition Module, QuantStudio Design & Analysis Software v1.5.1, FACSDiva 8.0) used for data collection in the paper are listed in the methods and are appropriately referenced.
Data analysis	Commercial software used for analysis (ImageJ v2.0.0, Keyence BZ-X700 Image Analyzer, CellRanger 4.0.0, QuantStudio Design & Analysis Software v1.5.1, ClampFit 11.0.3, Synaptosoft v6, NeuroScore v3.3.1, FACSDiva 8.0) are appropriately listed in the methods. No specific custom code was developed for any analyses. The analysis routine for single cell data is described in detail under 'Single cell RNA sequencing and analysis' and further details, if needed, will be provided upon request.

For manuscripts utilizing custom algorithms or software that are central to the research but not yet described in published literature, software must be made available to editors and reviewers. We strongly encourage code deposition in a community repository (e.g. GitHub). See the Nature Portfolio guidelines for submitting code & software for further information.

Data

Policy information about availability of data

All manuscripts must include a data availability statement. This statement should provide the following information, where applicable:

- Accession codes, unique identifiers, or web links for publicly available datasets
- A description of any restrictions on data availability
- For clinical datasets or third party data, please ensure that the statement adheres to our policy

Raw scRNAseq data that support the findings of this study have been deposited in the GEO Repository with the primary accession codes (GSE174417; https://www.ncbi.nlm.nih.gov/geo/query/acc.cgi?acc=GSE174417). Source data are provided with this paper. All other data are available as source data or from the

corresponding author upon reasonable request.

Field-specific reporting

Please select the one below that is the best fit for your research. If you are not sure, read the appropriate sections before making your selection.

☒ Life sciences ☐ Behavioural & social sciences ☐ Ecological, evolutionary & environmental sciences

For a reference copy of the document with all sections, see nature.com/documents/nr-reporting-summary-flat.pdf

Life sciences study design

All studies must disclose on these points even when the disclosure is negative.

Sample size	Sample sizes were not pre-determined. Sample size for electrophysiology was chosen based on previous experience with similar studies in pups (PMID: 31025941), while limiting use of animals and resources to support conclusions listed in the paper. In the case of in vivo behavioral assays, pre-experimentation power analysis was used to determine adequate sample size. Sample sizes are indicated where appropriate in text, methods, and/or legends.
Data exclusions	As described in the 'Single cell RNA sequencing and analysis' section, poor quality single cells were excluded based on the following parameters: UMIs per cell less than 1,500 or greater than 25,000; genes per cell < 1,000 or greater than 8,000; percentage of counts assigned to mitochondrial genes greater than 15%; more than 20 counts of hemoglobin genes per cell. No other data was excluded.
Replication	All experimental approaches used at least 3 biological replicates in summary data. Specific number of animals and trials are detailed in text, methods, and/or legends.
Randomization	Mice were randomly assigned to experimental and control groupings.
Blinding	Preparation of single cell suspensions for scRNAseq was not blind to genotype, in an effort to decipher if there were any changes in cell types of interest between cohorts. Since there were no decipherable differences between runs/control mice (as described in the 'Single cell RNA sequencing and analysis' section of the Methods, all individual runs were pooled for analysis. For all other experimentation, data collection and analysis was performed blind and then grouped based on genotype for statistical testing.

Reporting for specific materials, systems and methods

We require information from authors about some types of materials, experimental systems and methods used in many studies. Here, indicate whether each material, system or method listed is relevant to your study. If you are not sure if a list item applies to your research, read the appropriate section before selecting a response.

Materials & experimental systems

n/a	Involved in the study	
☐	☒	Antibodies
☒	☐	Eukaryotic cell lines
☒	☐	Palaeontology and archaeology
☐	☒	Animals and other organisms
☒	☐	Human research participants
☒	☐	Clinical data
☒	☐	Dual use research of concern

Methods

n/a	Involved in the study	
☒	☐	ChIP-seq
☐	☒	Flow cytometry
☒	☐	MRI-based neuroimaging

Antibodies

Antibodies used	Primary antibodies: Goat anti-human/mouse Phox2b, 1:100, R&D Systems, AF4940; Mouse anti-mouse somatostatin, 1:100, Santa Cruz Biotech, sc-55565; Rabbit-anti Lucifer yellow, 1:500, ThermoFisher, A-5750<br><br>Secondary antibodies (all 1:500 dilution, Jackson ImmunoResearch): Donkey anti-goat Cy3, 705-165-147; Donkey anti-mouse AlexaFluor 647, 715-605-150; Donkey anti-rabbit AlexaFluor 488, 711-545-152
Validation	Goat anti-human/mouse Phox2b antibody has been validated for use in ICC and ELISA as indicated on manufacturer's website https://www.rndsystems.com/products/human-mouse-phox2b-antibody_af4940. We have previously used this antibody for IHC applications (PMID: 31025941).<br><br>Mouse anti-mouse somatostatin has been validated for use in WB, IP, IF, IHC(P) and ELISA applications as indicated on the manufacturer's website: https://www.scbt.com/p/somatostatin-antibody-g-10<br><br>Rabbit-anti lucifer yellow antibody has extensive literature in amplification of lucifer yellow signal in a variety of preparations,

including post-hoc immunohistochemistry from patched neurons, as indicated on the manufacturer's website: https://www.thermofisher.com/antibody/product/Lucifer-Yellow-Antibody-Polyclonal/A-5750

Secondary antibodies from Jackson ImmunoResearch have been extensively used throughout life science literature: secondary antibodies that we selected were also tested for any cross reactivity in Bov, Ck, Gt, GP, Sy Hms, Hrs, Hu, Ms, Rat, Shp Sr Prot. More information can be found here: https://www.jacksonimmuno.com/technical/products/groups/whole-igg

Animals and other organisms

Policy information about studies involving animals; ARRIVE guidelines recommended for reporting animal research

Laboratory animals	P0-P45 male and female mice were used in equal proportions throughout experimentation. Specific age ranges per experimental paradigm are detailed in text, methods, and/or legends as appropriate. All animals were housed in a 12:12 light/dark cycle (average ambient temperature 72°F, average humidity 50%) with unlimited access to normal chow and an enrichment hutch. No other items were placed in home cages. Phox2bCre::TdT (Ai14) (JAX # 016223 and 007914) were crossed to Tcf4tr/+ mice to quantify aberrant Scn10a expression in the RTN and for anterograde tracing experiments. Tcf4tr/+ mice were also crossed with GfapCre/ERT2 (JAX # 012849) to assess parafacial astrocyte properties in this model. The Tcf4tr/+ line (JAX # 013598) used in this study was inbred from the F1 generation at time of cryorecovery and maintained on a 50% 129S1/SvlmJ, 50% C57BL6/J mixed background. There were no alterations in background strain throughout breeding or between different breeding schemes. Pups (mice ~P21 and below) were housed with both parents before experimentation and, in the case of in vivo experiments, during drug incubation periods between experiments. No pup was out of the home cage for more than 1 hour during in vivo trials.
Wild animals	No wild animals were used in this study.
Field-collected samples	No field-collected samples are used in this study.
Ethics oversight	University of Connecticut Institutional Animal Care and Use Committee (IACUC)

Note that full information on the approval of the study protocol must also be provided in the manuscript.

Flow Cytometry

Plots

Confirm that:

- [x] The axis labels state the marker and fluorochrome used (e.g. CD4-FITC).
- [x] The axis scales are clearly visible. Include numbers along axes only for bottom left plot of group (a 'group' is an analysis of identical markers).
- [x] All plots are contour plots with outliers or pseudocolor plots.
- [x] A numerical value for number of cells or percentage (with statistics) is provided.

Methodology

Sample preparation	Sample preparations for pooled qPCR are detailed in the methods section 'Florescence-activated cell sorting (FACS) and qRT-PCR' and as previously described (PMID: 32924935). In short, only adult (P30+) Phox2b-Cre:TdT:Tcf4tr/+ and Phox2b-Cre:TdT:Tcf4+/+ mice were used to make single cell suspension of all cells from the RTN, including TdT+ Phox2b RTN neurons. For each genotype, 3 animals were used to satisfy biological replicates; 3 technical replicates per sample were used for each Taqman probe.
Instrument	BD FACSAria II Cell Sorter
Software	BD FACSDiva 8.0
Cell population abundance	Phox2b (TdT+) cells made up about 21% of the total parent population of live (DAPI negative) cells in a 10,000 event sample (see Supplemental Information).
Gating strategy	Gating strategies are previously described in detail (PMID: 32924935). In short, a 10k threshold was applied to filter debris and dead cells from single cell suspensions. Single cells were then selected bsaed on side and forward scatter, and then gated on the absence of DAPI. Finally, cells were gated on the presence of dTomato fluorescence in 4-way purity mode.

- [x] Tick this box to confirm that a figure exemplifying the gating strategy is provided in the Supplementary Information.
